# Impact analysis of digital trade on carbon emissions from the perspectives of supply and demand

**DOI:** 10.1038/s41598-024-65658-1

**Published:** 2024-06-24

**Authors:** Huayou Zhu, Weiping Bao, Manman Qin

**Affiliations:** College of Economics and Management, Zhejiang Normal University, Jinhua City, 321004 Zhejiang Province China

**Keywords:** Digital trade, Carbon emissions, Green technological innovation, Consumption structure upgrading, Spatial spillover effect, Environmental economics, Environmental impact

## Abstract

Amidst the escalating challenge of global climate change, it is imperative to further explore whether digital trade, as an emerging element in the global development landscape, can reduce carbon emissions and achieve sustainable development. This study draws upon panel data encompassing 30 provinces and municipalities in China spanning the years 2013 to 2021. By establishing an index system to gauge regional digital trade development levels, the article examines the impact mechanism and spillover effects of digital trade on carbon reduction from both the supply (enterprises) and demand (residents) perspectives. The research results show that: (1) Digital trade can effectively promote regional carbon reduction, with a more pronounced effect in China's central and western regions and lower carbon emissions regions. (2) Digital trade can incentivize green innovation by enterprises and improve residents' consumption behavior, thereby reducing carbon emissions. (3) Digital trade has spillover effect on carbon emissions, and this “neighborhood effect” is greater than the “local effect”. Digital trade provides strong support for carbon reduction and sustainable development and also provides a strategic direction for government policy formulation.

## Introduction

In the past few years, the world economy has witnessed significant expansion, concurrently giving rise to urgent global issues such as environmental pollution and the escalating threat of global warming. Data from 2020 reveals that global carbon dioxide emissions constituted a substantial proportion of greenhouse gas emissions, reaching 72%, with a notable upward trajectory over recent decades^1^. As the largest global carbon emitter, China has assumed significant international responsibilities. In 2020, China outlined the enduring goals of reaching the peak of carbon emissions and achieving carbon neutrality by 2030 and 2060, respectively. This established low-carbon development as a crucial national strategic objective. The mitigation of carbon dioxide emissions is acknowledged as a central challenge for the global economy in addressing the impacts of global climate change.

Simultaneously, the interdisciplinary convergence of cutting-edge technologies, including big data analytics, artificial intelligence, and cloud-based solutions, has given rise to the phenomenon of digital trade. Digital trade encompasses the exchange of goods and services facilitated over the internet, prominently featuring digital content services, social networking platforms, search engines, various digital services, and e-commerce transactions^2^. Digital trade is characterized by three main attributes: Firstly, it operates on internet-based technology and is facilitated through online channels; Secondly, both parties involved in transactions digitize the transmission and delivery of data information; And finally, digital trade encompasses a broad spectrum, including both goods and services. Compared to traditional trade, digital trade drives the restructuring and transformation of traditional trade methods, trade partners, and trade entities, giving rise to new formats and models of trade. The advent of digital trade brings fresh opportunities for high-quality economic development and presents a potential avenue for carbon reduction.

Traditional trade is constrained by temporal and spatial limitations, necessitating both production and transportation to achieve physical delivery. In contrast, digital trade operates within the realm of the digital economy, harnessing the power of the internet and digital technologies to curtail transaction costs and energy consumption, thereby manifesting environmentally sustainable characteristics. Ma et al.^3^ indicates that trade behavior in the digital economy can reduce carbon emissions. In fact, many scholars have confirmed the emission reduction characteristics of digitization and digital technologies. Zhou et al.^4^ found that the use of information and communication technology can reduce energy input in production. The development of digitization optimizes factor allocation, contributing to increased energy efficiency and reduced carbon emissions^5,6^. Additionally, digitization can be applied to various aspects of the supply chain, including production, transportation, consumption, and recycling, to some extent alleviating the increase in carbon emissions caused by transportation^7^. However, considering that the use and upgrading of digital technologies require energy and power support, there may be a rebound effect leading to an increase in carbon emissions^8^. Zhou et al.^9^ found through the construction of a research framework on the carbon impact of digitization that carbon emissions resulting from digital demand and supply account for a significant share of total emissions. Therefore, there is a need for in-depth research into the carbon impact of digital trade in this digitized context.

The pertinent literature for this research falls within two primary domains: digital trade and carbon emissions. Firstly, in the realm of carbon emissions research, a predominant focus lies on measuring regional carbon emissions and their determinants^10–12^. Noteworthy factors such as industrial structure, economic output, population size, energy structure, and energy intensity emerge as key drivers of regional carbon emissions, offering valuable insights for selecting control variables in this study. Secondly, concerning the quantitative assessment of digital trade, the majority of scholars opt for constructing comprehensive indicator systems^13,14^. Additionally, given that digital trade hinges on transactions facilitated by ICT technology, a few scholars employ the trade value of ICT products and services as a proxy variable^15^. Current research predominantly focuses on the potential economic impacts of digital trade, encompassing aspects such as regional economic growth and market openness^16,17^. With regard to the research on the impact on carbon emission, based on the framework established by Grossman & Krueger^18^, and its impact mechanism is explored from the supply side, including scale effects, structural effects, and technological innovation effects^19–21^. In fact, these mechanisms primarily involve corporate behavior, but this evidently has certain limitations. Both enterprises and consumers, as dual participants in trade, are to some extent affected by digital trade, with changes in consumer behavior directly impacting carbon footprints. Therefore, it is crucial to focus on the carbon impact of digital trade from the demand perspective. Additionally, despite extensive literature discussing the spatial spillover effects of the digital economy on carbon emissions, the field of digital trade remains insufficiently addressed. Hence, there is a need to explore whether digital trade also generates similar spillover effects on carbon reduction.

Based on this, this paper delves into the mechanisms underlying the impact of digital trade on carbon emissions by employing a two-way fixed effects model, adopting a dual perspective that encompasses both supply and demand dynamics. Furthermore, this study employs spatial econometric models to investigate the spatial spillover effects of digital trade on carbon emissions. The overarching goal is to furnish insightful perspectives on the trajectory of low-carbon development, drawing from the realms of digital trade, green technological innovation, and the evolution of consumption structures. The primary contributions of this paper can be outlined as follows: (1) The study found that the occurrence of digital trade not only incentivizes green innovation among businesses but also improves residents' consumption behavior, both of which contribute to reducing carbon emissions. This provides new explanation for the environmental sustainability of digital trade. (2) Previous research has mainly focused on how digital trade affects carbon emissions, with a lack of further research on its spillover effects. This study constructed a spatial durbin model based on the network characteristics of digital trade to explore the spillover effects of digital trade on carbon emissions between regions, thereby enriching the research content of digital trade.

## Theoretical mechanisms and hypotheses

### The impact of digital trade on carbon emissions

Previous research has shown that the scale effect observed in traditional trade tends to intensify the input of production factors and energy consumption, ultimately leading to a surge in carbon emissions and worsening environmental degradation^22,23^. Compared to traditional trade, digital trade, due to its characteristics such as virtualization and platformization, has the potential to reduce carbon emissions.

On one hand, digital trade involves trade in digitized products and services that can be transmitted through information and communication technology. In the trading process, digital trade can effectively reduce transaction costs for businesses, decrease energy consumption during the transportation of goods, and mitigate the continuous rise in carbon emissions^19,21^. Moreover, digital trade includes digital services trade, and its development is likely to promote the growth of other service industries such as e-commerce, leading to the shift from the secondary sector to the tertiary sector. These industries have environmentally friendly characteristics and contribute less to environmental pollution, pressuring energy-intensive and highly polluting industries to undergo digital transformation^24^. Consequently, it fosters the growth of green and low-carbon industries, ultimately leading to a reduction in carbon emissions^25^.

On the other hand, traditional trade processes require specific trading venues, cumbersome transaction procedures, and paper legal documents. In contrast, digital trade is conducted on internet platforms such as Taobao and JD.com, which greatly improves trade efficiency^26^. With technologies such as information communication and blockchain, platform-based transactions are now more transparent and efficient, facilitating information exchange between trading parties. This leads to a substantial decrease in information search and transmission costs for trading entities, effectively addressing the challenge of information asymmetry between the supply and demand sides^27^.

Building upon these considerations, this paper proposes the following hypotheses:

#### Hypothesis 1

Digital trade can inhibit the increase in regional carbon emissions.

### The mechanisms of digital trade on carbon emissions

**Figure 1:**
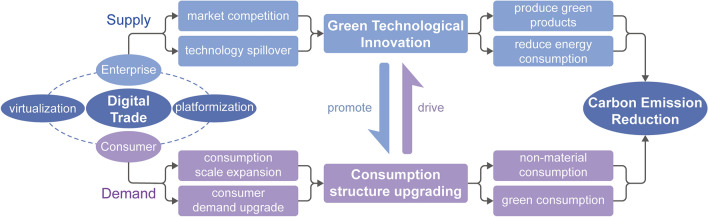
Diagram of influence mechanism.

The rise of digital trade has far-reaching implications for both trading parties, namely enterprises and consumers^28^. Hence, the primary focus of this paper is to delve into the mechanisms of how digital trade influences carbon emissions, considering the dual perspectives of both supply and demand, as illustrated in Fig. 1.

#### From the supply perspective

The development of rules and systems in digital trade will place higher demands on digital technology and traded products. Concurrently, enterprises, aiming for expanded market shares, will consistently escalate investments in technological research and development. This endeavor is undertaken to attain green technological innovation, ensuring the sophistication and environmental sustainability of both technology and products^29^. Technologies like internet cloud computing and big data can effectively disclose information about the production processes of enterprises, facilitating government supervision and governance. These external regulatory pressures will motivate enterprises to accelerate their pace of green technological innovation. Moreover, digital products often entail technological spillovers during trade, enabling companies to learn and absorb advanced technologies from external sources, leading to innovation^19,30^.

Meanwhile, green technological innovation can effectively address environmental pollution. Through green technological innovation, enterprises can improve energy efficiency and produce green products^31^, while residents can enhance energy conservation and increase green consumption. Ultimately, this culminates in a significant reduction in carbon emissions.

#### From the demand perspective

Digital trade can effectively stimulate consumption growth and an upgrade in consumption demand. On one hand, digital trade incorporates user-friendly mobile payment options, fosters mutual trust among transaction participants, and showcases a network effect, all contributing to the effective stimulation of residents' consumption expenditures^32,33^. On the other hand, digital trade has spawned various innovative trade formats and models, resulting in progressively personalized and virtualized trade content and diverse, intelligent trading methods. This has encouraged residents to shift from subsistence-oriented consumption to development and enjoyment-oriented consumption, reflecting an upgrade in consumption demand^21^.

Changes in residents' consumption patterns play a crucial role in driving the reduction of carbon emissions^34,35^. On one side, the upgrading of consumption patterns encourages residents to seek elevated non-material consumption experiences and amplifies the share of the service industry in overall consumption^21,36^. It also promotes the widespread dissemination of green consumption awareness, as environmentally conscious residents are more inclined to purchase green products and services, thereby curbing the rapid growth of carbon emissions. On the flip side, changes in consumption structure ultimately affect industrial structure, leading to an upgrade in industrial structure^37^. The heightened green consumption awareness of residents will drive enterprises to undergo green transformation through demand feedback effects, resulting in the production of more green products^38^. Consequently, this contributes to a reduction in both energy consumption and carbon emissions.

Building upon these considerations, this paper proposes the following hypotheses:

##### Hypothesis 2

Digital trade can promote carbon emission reduction through the dual pathways of green technological innovation and the consumption structure upgrading.

### Spatial spillover effects of digital trade on carbon emissions

Due to the presence of network externalities, economic activities can extend their impact beyond local areas, creating spatial spillover effects that influence neighboring regions. The network infrastructure inherent in digital trade demonstrates robust spillover effects, impacting economic variables not only within its own region but also in adjacent areas^39^. Moreover, artificial intelligence and big data, as digital technologies, have the potential to generate cascading impacts in proximate areas via interconnected networks, cooperative business endeavors, and the establishment of platform economies^40,41^. Additionally, Studies indicate that carbon emissions in separate geographical areas can mutually influence one another, showcasing spatial effects in the intensity of carbon emissions^42^. And the restraining impact of the digital economy on regional carbon emissions is evident at both local and neighboring levels^41^.

Building upon these considerations, this paper proposes the following hypotheses:

#### Hypothesis 3

The impact of digital trade on regional carbon emissions exhibits spatial spillover effects.

## Methods

### Model specification

To assess the potential impact of digital trade on carbon emissions, this research employs empirical analysis, employing regional carbon emissions as the dependent variable and the degree of digital trade development as the explanatory variable. The econometric model designed for this study is structured as follows:

1 $$\text{ln}\, {ce}_{it}=\alpha +{\alpha }_{1}{dt}_{it}+{\alpha }_{2}{X}_{it}+{u}_{i}+{v}_{t}+{\varepsilon }_{it}$$

where ${ce}_{it}$ and ${dt}_{it}$ represent the carbon emission level and digital trade development level, respectively, and ${X}_{it}$ is a set of control variables. $\alpha$ represents the intercept term, ${\alpha }_{1}$ and ${\alpha }_{2}$ represent the regression coefficients of the explanatory variables and control variables, ${u}_{i}$ and ${v}_{t}$ represent regional and time fixed effects, and ${\varepsilon }_{it}$ represents the error term.

Additionally, to investigate the intermediate mediation mechanism of how digital trade suppresses the increase in carbon emissions, an intermediate variable is introduced based on the baseline model. The mediation effect model is shown as follows, with the intermediate variable *M* being green technological innovation (*gti*) and consumption structure upgrading (*csu*).

2 $$\text{ln}\,{ ce}_{it}=\alpha +{\beta }_{1}{dt}_{it}+\gamma {X}_{it}+{u}_{i}+{v}_{t}+{\varepsilon }_{it}$$

3 $${M}_{it}=\alpha +{\beta }_{2}{dt}_{it}+\gamma {X}_{it}+{u}_{i}+{v}_{t}+{\varepsilon }_{it}$$

4 $$\text{ln}\, {ce}_{it}=\alpha +{\beta }_{1}{dt}_{it}+{\beta }_{2}{M}_{it}+\gamma {X}_{it}+{u}_{i}+{v}_{t}+{\varepsilon }_{it}$$

Furthermore, introducing spatial lag terms for each variable and employing spatial econometric models, this study further verifies whether digital trade can promote carbon emission reduction while exploring possible spatial spillover effects. The spatial econometric model is as follows:

5 $$\text{ln}\,{ ce}_{it}=\alpha +\rho \sum_{i=1}^{n}{W}_{ij}ln\,{ ce}_{it}+{\alpha }_{1}{dt}_{it}+{\theta }_{1}\sum_{i=1}^{n}{W}_{ij}{dt}_{jt}+{\alpha }_{2}\sum {X}_{it}+{\theta }_{2}\sum_{i=1}^{n}{w}_{ij}{X}_{ji}+{u}_{i}+{v}_{t}+{\varepsilon }_{it}$$

where $\rho$ and $\theta$ represent the spatial autoregressive coefficient and spatial lag term coefficients, while the other variables remain consistent with the previous description. ${W}_{ij}$ is the spatial weight matrix. Following the method by Gan et al.^43^, this paper primarily uses the following spatial weight matrices. First, the adjacency matrix ${W}_{1}$ is constructed using 0 and 1 to represent the adjacency between regions, with 1 indicating adjacency and 0 indicating non-adjacency. Second, the geographical distance spatial weight matrix ${W}_{2}$ is constructed based on the reciprocal of the distance ${d}_{ij}$ between regional capitals calculated using latitude and longitude. Third, the economic geography spatial weight matrix ${W}_{3}$, as shown in Eq. 6. Here, ${X}_{i}$ and ${X}_{j}$ respectively signify the average per capita gross domestic product of provinces i and j throughout the sample period.

6 $${W}_{3}=\left\{\begin{array}{l}\frac{1}{|{X}_{i}-{X}_{j}|}\times \frac{1}{{d}_{ij}}, i\ne j\\ 0, i=j\end{array}\right.$$

### Variable measurement

#### The dependent variable

Carbon emission level (ln_ce). Following the method proposed by Chen et al.^44^, the total carbon emissions (ce) for each region are calculated based on the consumption of the eight major fossil fuels (c), using standard coal conversion coefficients (scc), and carbon emission coefficients (cec) provided by IPCC^45^. The carbon emission level is then obtained by taking the logarithm of the calculated carbon emissions. The specific formula for calculating carbon emissions is as follows:

7 $$ce=\sum_{i=1}^{8}{c}_{i}*{scc}_{i}*{cec}_{i}*\frac{44}{12}$$

#### The explanatory variable

Digital Trade (dt). Combining the China digital trade indicator system proposed by Ma et al.^46^ and the research of scholars such as Hu et al.^13^, this study constructs a regional digital trade development level indicator system, which includes four dimensions: Digital trade market potential, Digital trade structure, Digital trade environment, and Digital trade technical support. The specific details are shown in Table 1.

**Table 1:** Comprehensive index of regional digital trade development.

Dimension	Secondary indicator	Tertiary indicator	Weight
Digital trade market potential	Trade openness	Total import and export value/regional gdp	0.041
	Residential consumption potential	Per capita residential consumption expenditu	0.022
		Retail sales of consumer goods	0.033
Digital trade structure	Digital product structure	ICT product exports/total product exports	0.026
	Digital service structure	ICT service exports/total service exports	0.026
		Digital service form exports/total service exports	0.027
Digital trade environment	Digital infrastructure	Number of domains	0.064
		Number of webpages	0.114
		Number of Internet access ports	0.029
		Length of long-distance fiber optic cables	0.020
	Digital industrialization	Telecommunications business	0.061
		Software business revenue	0.093
		Information technology service revenue	0.095
	Industrial digitalization	Number of computers per 100 employees in enterprises	0.022
		E-commerce sales volume	0.067
		Proportion of e-commerce transaction-active enterprises	0.014
	Digital trade security	Patent non-infringement rate	0.028
		Public safety budget expenditure	0.026
Digital trade technical Support	Digital technology talent	Employment of Urban Units in Information Transmission, Software, and Information Technology Services	0.059
	innovation capability	R&D Expenditure	0.062
		Number of invention patent applications and authorizations	0.071

The weight is calculated according to the entropy method.

Based on this indicator system, the entropy weight method was employed to assess the level of digital trade in different regions of China. The calculation method for the entropy method is as follows:

8 $${x}_{ij}^{0}=\frac{{x}_{ij}-min({x}_{ij})}{max({x}_{ij})-min({x}_{ij})}$$

9 $${p}_{ij}=\frac{{x}_{ij}^{0}}{\sum_{i=1}^{n}{x}_{ij}^{0}}$$

10 $${e}_{j}=-\frac{1}{ln(n)}\sum\limits_{i=1}^{n}{p}_{ij}ln({p}_{ij})$$

11 $${w}_{j}=\frac{1-{e}_{j}}{{\sum }_{j=1}^{m}{(1-e}_{j})}$$

12 $${dt}_{i}=\sum\limits_{j=1}^{m}{w}_{j}{x}_{ij}^{0}$$

Among them, *i* and *j* represent the year and item, respectively. Standardize the various indicators ${x}_{ij}$ in the index system to ${x}_{ij}^{0}$. Then, calculate the entropy values (${e}_{j}$) and weights (${w}_{j}$)of each indicator, and through computation, obtain the level of digital trade development (*dt*).

**Figure 2:**
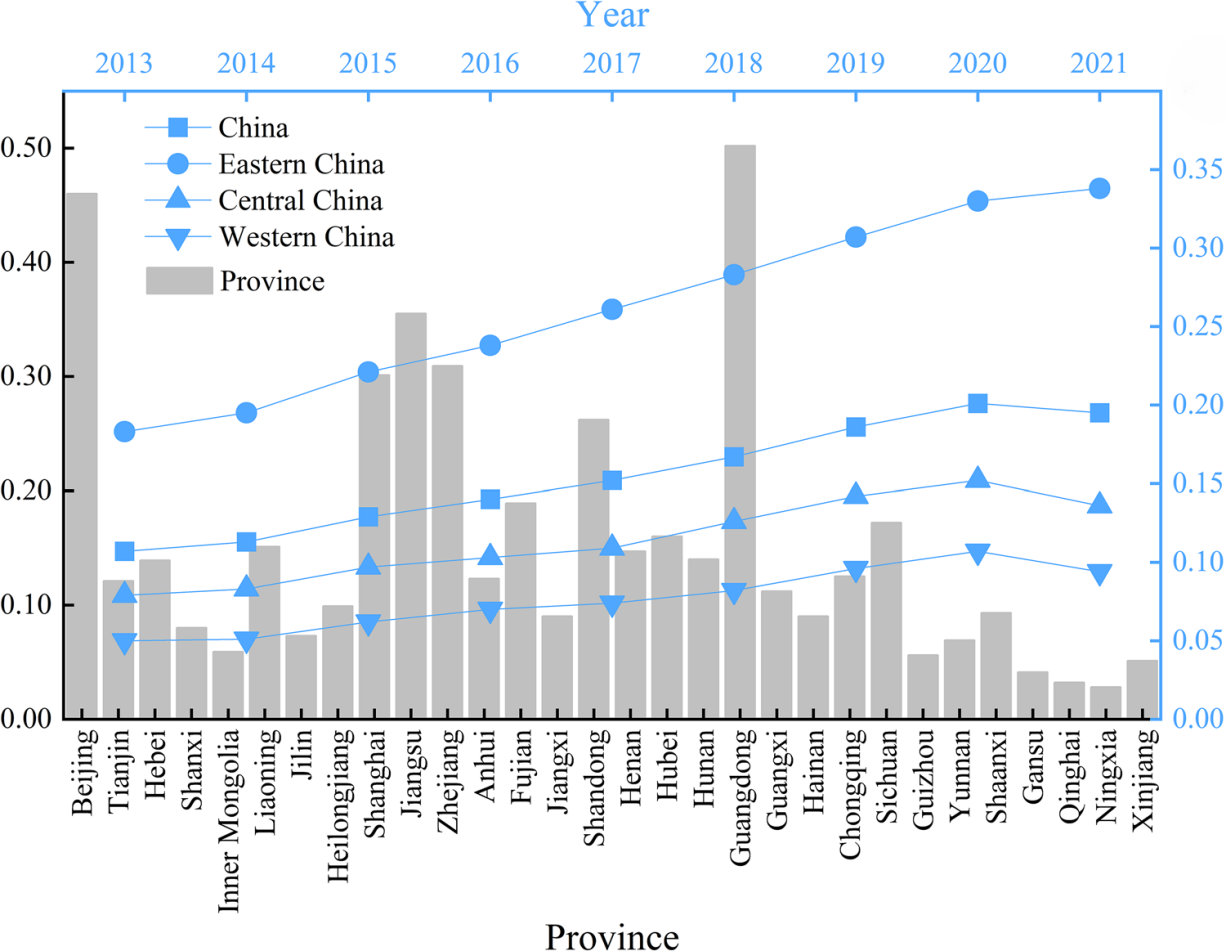
The development level of digital trade in China.

Figure 2 presents the development levels of digital trade in various regions and their temporal trends. Digital trade shows a characteristic of “year-on-year increase, gradually weakening from east to west”. Looking at the average values of digital trade during the sample period, the five regions with relatively high levels are Guangdong (0.502), Beijing (0.460), Jiangsu (0.355), Shanghai (0.301), and Zhejiang (0.309), all of which are in the eastern part of China. On the other hand, the five regions with relatively low levels of digital trade are Ningxia, Qinghai, Gansu, Xinjiang, and Guizhou, all located in the western part of the country. Regarding the temporal trends in digital trade, both the national and eastern, central, and western regions all showed an upward trend in digital trade. Among these regions, the eastern region exhibited the most favorable trend in digital trade development, boasting the highest growth rate. Meanwhile, the progress of digital trade in the central and western regions lagged behind, maintaining a level below the national average for digital trade development.

#### Mechanism variables

1. Green technology innovation (gti): Traditional methods for measuring technological progress often cannot distinguish between general technological advancements and green technologies. Therefore, following the approach of Feng et al.^47^, this study uses the number of green technology invention patents authorized to represent the level of green technology innovation.
2. Consumption structure upgrading (csu): The transformation of the consumption structure is evident in the shift in residents' spending patterns, specifically characterized by the proportion of expenditures on high-level consumption. Uses the Extended Linear Expenditure System (ELES) to calculate the income elasticity of demand for the eight major categories of residents' consumption expenditures. The formula is as follows:

13 $${c}_{i}={a}_{i}+{b}_{i}y+{\varepsilon }_{i}$$

14 $${EM}_{i}=\frac{\partial {x}_{i}/{x}_{i}}{\partial y/y}={b}_{i}\frac{y}{{x}_{i}{p}_{i}}={b}_{i}\frac{y}{{c}_{i}}$$

where ${c}_{i}$ represents the total expenditure on the i-th commodity, y stands for residents’ disposable income, ${b}_{i}$ denotes the marginal consumption propensity of the i-th commodity, and the corresponding ${EM}_{i}$ represents the income elasticity of demand. The calculation results are as shown in Table 2. The average income elasticity of demand for various consumption expenditures is 0.835. Consumption expenditures with income elasticity of demand greater than the average (residential, transportation and communication, education, culture and entertainment, and healthcare expenditures) are categorized as high-level consumption. This study uses the proportion of high-level consumption in residents' consumption expenditures as a proxy variable for the upgrading of consumption structure.

**Table 2:** Income elasticity of various categories of residents' consumption expenditures.

Year	Food, tobacco and alcohol	Clothes	Housing	Household goods and services	Traffic and communication	Education, culture, and entertainment	Healthcare	Others
2013	0.761	0.348	1.083	0.798	0.912	0.836	1.207	0.691
2014	0.769	0.359	1.117	0.796	0.875	0.838	1.160	0.690
2015	0.782	0.369	1.139	0.811	0.853	0.813	1.134	0.691
2016	0.793	0.387	1.127	0.802	0.826	0.794	1.095	0.718
2017	0.829	0.410	1.121	0.814	0.843	0.794	1.076	0.712
2018	0.859	0.428	1.077	0.811	0.855	0.809	1.007	0.724
2019	0.866	0.449	1.078	0.843	0.871	0.780	0.971	0.718
2020	0.863	0.508	1.094	0.898	0.945	1.011	1.050	0.853
2021	0.839	0.484	1.104	0.867	0.902	0.862	0.998	0.756
Average	0.818	0.416	1.105	0.827	0.876	0.838	1.077	0.728

#### Control variables

Drawing from relevant research^48–50^, this study includes the following control variables: (1) Industry structure (istr), expressed as the proportion of value-added in the tertiary industry relative to that in the secondary industry. (2) Population size (ln_pop), characterized by the logarithm of the annual resident population. (3) Urbanization (urban), indicated by the proportion of the urban population. (4) Foreign direct investment (fdi), represented by the ratio of foreign direct investment to regional gross domestic product. (5) Government intervention (gov), signified by the ratio of government fiscal expenditures to regional gross domestic product. (6) Energy consumption (ln_ec), represented by the logarithm of the annual electricity consumption. (7) Energy structure (estr), depicted by the ratio of coal consumption to total energy consumption in each region.

### Data sources

Given the relatively brief evolution of digital trade and the challenge of incomplete data, this research made use of panel data extracted from 30 provinces within China, excluding Tibet, Hong Kong, Macao, and Taiwan. The data spanned the years 2013 to 2021 and was predominantly sourced from reputable outlets, including the "China Statistical Yearbook," various regional statistical yearbooks, the "China Energy Statistical Yearbook," the "China Commerce Yearbook," the "China Trade and Foreign Economic Statistical Yearbook," the National Bureau of Statistics, and the State Intellectual Property Office. Table 3 presents descriptive statistics for the variables under consideration. Additionally, this study conducted multicollinearity tests on the variables, as shown in Table 4, where the variance inflation factor (VIF) for control variables is less than 10.

**Table 3:** Descriptive statistics.

Variable attributes	Variable	Obs	Mean	Std. Dev	Min	Max
Explained variable	ln_ce	270	10.508	0.785	8.575	12.378
Explanatory variable	dt	270	0.154	0.128	0.015	0.691
Mechanism variables	gti	270	0.307	0.572	0.122	4.573
	csu	270	0.305	0.037	0.250	0.494
Control variables	istr	270	1.410	0.744	0.665	5.244
	ln_pop	270	8.212	0.741	6.347	9.448
	urban	270	0.609	0.115	0.379	0.896
	fdi	270	0.176	0.016	0.000	0.121
	gov	270	0.263	0.111	0.107	0.753
	ln_ec	270	7.460	0.684	5.447	8.970
	estr	270	0.920	0.485	0.011	2.549

**Table 4:** Multicollinearity tests.

Variable	istr	In_pop	urban	fdi	gov	ln_ec	estr
VIF	2.62	8.54	4.27	1.32	4.38	5.20	1.77
1/VIF	0.382	0.117	0.234	0.757	0.228	0.192	0.565

## Empirical analysis

### Baseline regression

The estimated impact of digital trade on carbon emission levels is shown in Table 5. Column (5) demonstrates that, with the simultaneous introduction of control variables and controlling for provincial and time fixed effects, the regression results show that the coefficient of digital trade is negative at the 1% significance level. This suggests that the development of digital trade can effectively curb the increase in carbon emission levels, consistent with the findings of Wang et al. (2023) and Ji et al. (2023), with estimated coefficients also falling within a reasonable range, thereby supporting Hypothesis 1.

**Table 5:** Baseline regression results.

Variable	(1)	(2)	(3)	(4)	(5)
dt	0.826**	− 1.043***	− 0.430***	− 0.443**	− 0.660***
	(0.371)	(0.237)	(0.134)	(0.171)	(0.172)
intr		0.036	− 0.107***		− 0.159***
		(0.028)	(0.036)		(0.045)
ln_pop		0.462***	1.219***		1.421***
		(0.051)	(0.218)		(0.251)
urban		1.779***	− 0.847***		− 1.669***
		(0.232)	(0.274)		(0.474)
fdi		1.291	− 1.363***		− 0.957*
		(0.901)	(0.454)		(0.500)
gov		0.413*	0.498**		0.654**
		(0.242)	(0.223)		(0.262)
ln_ec		0.572***	0.625***		0.561***
		(0.043)	(0.066)		(0.085)
estr		0.954***	0.399***		0.391***
		(0.035)	(0.057)		(0.058)
_cons	10.380***	0.469	− 3.909**	10.525***	− 4.611**
	(0.074)	(0.479)	(1.762)	(0.025)	(1.847)
region-effect	NO	NO	YES	YES	YES
time-effect	NO	NO	NO	YES	YES
adj. R^2^	0.014	0.928	0.481	0.006	0.480
N	270	270	270	270	270

(1) ***, **, and * represent statistical significance levels at 1%, 5%, and 10%, respectively. (2) t statistics are in parentheses.

As one of the world's largest manufacturing and trading centers, China has historically incurred significant energy consumption and carbon emissions due to traditional trade production and transportation processes. The emergence of digital trade invariably fosters enhanced efficiency in product supply chain management, logistics, and transportation systems, thereby alleviating the escalation of carbon emissions.

### Robustness tests

In order to augment the credibility of the baseline regression outcomes, a series of examinations for robustness have been undertaken. (1) Replace the explained variable. This paper simultaneously takes the per capita carbon emissions(ln_ce1) obtained by dividing the total carbon emissions in the region by the total population, and the carbon emission intensity(ln_ce2) obtained by dividing the total carbon emissions in the region by the regional gross domestic product as the explained variables. (2) Trimming procedure. To eliminate the influence of outliers, this paper conducts a 1% trimming on the explained variable carbon emissions and the core explanatory variable digital trade before conducting the benchmark regression. (3) Cluster id. Regression analysis is conducted using stricter clustering to the individual level standard errors. (4) Eliminate samples. To avoid the possible influence of regional administrative nature, regression is conducted after excluding samples of the four municipalities directly under the central government: Beijing, Tianjin, Shanghai, and Chongqing. (5) 2SLS. Lagged one period and lagged two periods of digital trade are used as instrumental variables respectively, and regression is conducted using two-stage least squares (2SLS). (6) SYS-GMM. To avoid potential endogeneity, the System-GMM model is adopted, and the lagged one-period term of carbon emissions is included in the regression analysis.

The robustness analysis results are shown in Table 6 above. The estimated coefficient of the explanatory variable digital trade remains significantly negative, consistent with the benchmark regression results.

**Table 6:** Robustness tests.

Variable	(1)		(2)	(3)	(4)	(5)		(6)
	Replacement variables		Trimming	Cluster id	Eliminate samples	2SLS		SYS-GMM
	ln_ce1	ln_ce2	ln_ce	ln_ce	ln_ce	ln_ce	ln_ce	ln_ce
L. ln_ce								0.422***
								(0.061)
dt	− 0.660***	− 0.907***	− 0.686***	− 0.660*	− 0.645***	− 0.955***	− 0.593***	− 0.484***
	(0.172)	(0.204)	(0.173)	(0.331)	(0.194)	(0.206)	(0.214)	(0.179)
Control variables	YES	YES	YES	YES	YES	YES	YES	YES
region-effect	YES	YES	YES	YES	YES	YES	YES	YES
time-effect	YES	YES	YES	YES	YES	YES	YES	YES
adj. R^2^	0.409	0.850	0.457	0.993	0.558	0.630	0.540	0.565
N	270	270	270	270	234	240	210	240
LM statistic						167.678***	114.189***	
Wald statistic						772.588	288.025	
						[16.38]	[16.38]	
AR(2)* P* value								0.250

(1) ***, **, and * represent statistical significance levels at 1%, 5%, and 10%, respectively. (2) t statistics are in parentheses. (3) values in brackets are the critical values from the Stock-Yogo test at the 10% statistical significance level for weak instrument identification.

### Heterogeneity analysis

Given that the influence of digital trade on carbon emissions might exhibit variations among diverse regions, additional examination becomes imperative.

#### Geographic region heterogeneity analysis

China's mainland is geographically categorized into eastern and central-western regions, excluding Tibet, for the purpose of grouped regression. In Table 7, column (1) and (2), both the eastern and central-western regions display a notably negative inhibitory impact of digital trade on carbon emission levels. However, the magnitude of the impact coefficient of digital trade in the central-western region is 2.106, while it is merely 0.327 in the eastern region. This indicates a more pronounced carbon reduction effect of digital trade in China's central-western region. Potential reasons for this divergence include the advanced economic development and abundant talent and innovation resources in the Eastern region, leading to a higher level of digital trade development. Consequently, the marginal contribution of digital trade to carbon emissions reduction in this region is relatively constrained. Additionally, the unregulated expansion of the digital trade scale may result in resource wastage and an increase in carbon emissions. In contrast, the less developed central-western region of China leverages the "latecomer advantage" of digital trade. It can optimize its industrial structure, upgrade technology, enhance energy efficiency, and facilitate the reduction of carbon emissions.

**Table 7:** Heterogeneity analysis.

Variable	(1)	(2)	(3)	(4)
	Eastern region	Central-western region	High carbon region	Low carbon region
dt	− 0.327*	− 2.106***	− 0.388*	− 0.940***
	(0.196)	(0.450)	(0.219)	(0.256)
Control variables	YES	YES	YES	YES
Region-effect	YES	YES	YES	YES
Time-effect	YES	YES	YES	YES
Adj. R^2^	0.619	0.522	0.703	0.454
N	99	171	135	135

(1) ***, **, and * represent statistical significance levels at 1%, 5%, and 10%, respectively. (2) t statistics are in parentheses.

#### Carbon emission heterogeneity analysis

Utilizing the computed carbon emission levels across different regions in 2021, classified based on the median, regions are grouped into high-carbon emission and low-carbon emission areas for the purpose of grouped regression. As illustrated in Table 7, column (3) and (4), the impact coefficients of digital trade in both high-carbon emission and low-carbon emission areas exhibit significant negativity. However, the absolute values of these impact coefficients suggest a more pronounced inhibitory effect of digital trade on carbon emissions in low-carbon emission areas. This observation may be attributed to the characteristics prevalent in most of China's high-carbon emission areas, such as a singular energy structure and high energy consumption, making it challenging to swiftly achieve short-term carbon reduction goals. Consequently, the inhibitory effect of digital trade in these regions may be somewhat constrained, necessitating long-term and sustained efforts for effective carbon reduction.

### Mechanism analysis

To investigate whether there are mechanisms in both the supply and demand aspects of digital trade that promote carbon reduction, we introduced intermediate variables: green technology innovation and consumption structure upgrade. We conducted mechanism analysis using a stepwise regression approach, and the results are shown in Table 8.

**Table 8:** Mechanism analysis.

Variable	(1)	(2)	(3)	(4)
	gti	csu	ln_ce	ln_ce
dt	2.917***	0.053**	− 0.448**	− 0.608***
	(0.291)	(0.024)	(0.206)	(0.173)
gti			− 0.073*	
			(0.039)	
csu				− 0.982**
				(0.482)
Control variables	YES	YES	YES	YES
Region-effect	YES	YES	YES	YES
Time-effect	YES	YES	YES	YES
adj. R^2^	0.656	0.769	0.485	0.487
N	270	270	270	270

(1) ***, **, and * represent statistical significance levels at 1%, 5%, and 10%, respectively. (2) t statistics are in parentheses.

From the supply perspective, as evidenced in the initial column of Table 8, the impact coefficient of digital trade on the advancement of green technology innovation is notably positive, reaching a level of significance at 1%. This suggests that the expansion of digital trade holds the potential to stimulate innovation in environmentally friendly technologies within enterprises, leading to reduced energy consumption, the realization of clean production, and a decrease in carbon emissions. And from the demand perspective, as depicted in the second column of Table 8, the impact coefficient of digital trade on the enhancement of consumption structure registers significance at the 5% level. This implies that digital trade has the capacity to drive the refinement of residents' consumption patterns, increasing the share of high-level consumer expenditure among residents. Finally, a regression analysis was performed encompassing the independent variable, intermediary variables, and dependent variable. The outcomes in columns (3) and (4) of Table 8 reveal that the impact coefficients of digital trade and intermediary variables on carbon emissions are both significantly negative. Through a dual pathway involving enterprise green technology innovation and the upgrading of residential consumption structure, digital trade emerges as a driver for carbon reduction, thereby supporting Hypothesis 2.

### Analysis of spatial spillover effect

#### Spatial autocorrelation test

A crucial prerequisite for constructing spatial econometric models is the assumption that variables exhibit a certain degree of spatial autocorrelation. Currently, the most common method for testing this assumption is to use Moran's I index to analyze the spatial correlation and clustering characteristics of variables. The calculation is as follows:

15 $$I=\frac{n\sum_{i=1}^{n}\sum_{j=1}^{n}{W}_{ij}\left({X}_{i}-\overline{X }\right)\left({X}_{j}-\overline{X }\right)}{\sum_{i=1}^{n}{\left({X}_{i}-\overline{X }\right)}^{2}\sum_{i=1}^{n}\sum_{j=1}^{n}{W}_{ij}}$$

where ${X}_{i}$ and ${X}_{j}$ represent the carbon emissions or digital trade levels of regions *i* and *j*. The symbol $\overline{X }$ denotes the mean of ${X}_{i}$ or ${X}_{j}$, and ${W}_{ij}$ represents the spatial weight matrix. Moran’ s I index values fall within the range of − 1 to 1, and the magnitude indicates the degree of spatial correlation for the variable. Moran's I greater than 0 signifies positive spatial correlation in the variable *X*.

Global spatial autocorrelation analysis. As shown in Table 9, from 2013 to 2021, carbon emissions (*ce*) and digital trade(*dt*), under the conditions of spatial weight matrices ${\text{W}}_{1}$, ${\text{W}}_{2}$, and ${\text{W}}_{3}$, all exhibit positive global Moran's I indices at a significance level of at least 10%, and they display a positive spatial clustering distribution.

**Table 9:** Moran' s I values for carbon emissions and digital trade levels from 2013 to 2021.

Year	${\text{W}}_{1}$		${\text{W}}_{2}$		${\text{W}}_{3}$	
	ce	dt	ce	dt	ce	dt
2013	0.265***	0.219**	0.069***	0.045**	0.335***	0.252***
2014	0.242***	0.239**	0.065***	0.048**	0.329***	0.268***
2015	0.254***	0.226**	0.065***	0.041**	0.310***	0.260***
2016	0.242***	0.216**	0.059***	0.037**	0.286***	0.261***
2017	0.225**	0.209**	0.052***	0.036**	0.248***	0.262***
2018	0.218**	0.180**	0.056***	0.029**	0.276***	0.232***
2019	0.210**	0.147*	0.054***	0.022*	0.264***	0.214**
2020	0.203**	0.131*	0.052***	0.019*	0.248***	0.195**
2021	0.165**	0.131*	0.041**	0.018*	0.215***	0.210**

***, **, and * represent statistical significance levels at 1%, 5%, and 10%, respectively.

**Figure 3:**
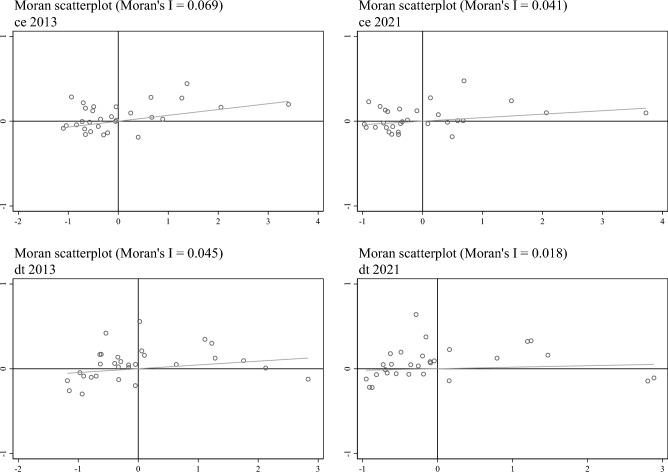
Moran scatter plots of carbon emissions and digital trade in 2013 and 2021.

Furthermore, using Moran scatter plots and selecting two representative years, 2013 and 2021, we focused on examining the local distribution of carbon emissions and digital trade levels in geographic space using the geographical distance weight matrix ${\text{W}}_{2}$, as shown in Fig. 3. Overall, regional carbon emissions (*ce*) and digital trade (*dt*) tend to concentrate predominantly in the first and third quadrants, demonstrating a clustering pattern of "high-high" and "low-low." This observation further underscores the notable spatial autocorrelation present in these variables.

#### Analysis of spatial dubin model regression results

After conducting LM tests, LR tests, Wald tests, and Hausman tests, as shown in Appendix 1, we opted for the spatial durbin model (SDM) with adjustments for fixed effects in both regional and temporal dimensions.

The regression outcomes from the model, as presented in Table 10, reveal that irrespective of whether the adjacency matrix, geographical distance spatial matrix, or economic geography spatial matrix is employed, the coefficient associated with digital trade on carbon emissions is consistently negative and statistically significant at the 1% level. This indicates that digital trade can effectively restrain the escalation of carbon emissions, thereby corroborating hypothesis 1. Furthermore, the significance of both spatial autocorrelation coefficients (ρ) and the spatial lag term of digital trade (W × dt) in the Spatial Durbin Model regression results suggests the presence of exogenous interactive effects of digital trade in spatial terms and endogenous interactive effects of carbon emissions. This preliminary evaluation hints at the potential existence of spatial spillover effects in the inhibitory impact of digital trade on carbon emissions.

**Table 10:** Results of the spatial dubin model regression.

Variable	(1)	(2)	(3)
	${\text{W}}_{1}$	${\text{W}}_{2}$	${\text{W}}_{3}$
dt	− 0.822***	− 0.941***	− 0.797***
	(0.177)	(0.196)	(0.175)
W × dt	− 1.026***	− 4.494***	− 1.193**
	(0.388)	(1.495)	(0.494)
Control variables	YES	YES	YES
Region-effect	YES	YES	YES
Time-effect	YES	YES	YES
$\uprho$	0.192**	− 0.459*	− 0.190*
	(0.087)	(0.271)	(0.101)
LR-direct effects	− 0.876***	− 0.867***	− 0.750***
	(0.199)	(0.194)	(0.176)
LR-indirect effects	− 1.399**	− 2.977**	− 0.893**
	(0.555)	(1.395)	(0.438)
LR-total effects	− 2.275***	− 3.843**	− 1.644***
	(0.707)	(1.508)	(0.511)
Log-likelihood	402.227	395.365	387.793
N	270	270	270

(1) ***, **, and * represent statistical significance levels at 1%, 5%, and 10%, respectively. (2) t statistics are in parentheses.

Adopting the approach outlined by Lesage & Pace^51^, the spatial inhibitory effect of digital trade on carbon emissions is deconstructed into direct effects, indirect effects, and total effects. The assessment of the significance of the indirect effects is performed to ascertain the presence of spatial spillover effects. In this context, the direct effect signifies the influence of local digital trade development on local carbon emissions, the indirect effect represents the impact of digital trade in neighboring regions on local carbon emissions, referred to as spatial spillover effects, and the total effect is the summation of the direct and indirect effects.

The findings suggest that the levels of digital trade development in both local and neighboring regions exert a substantial inhibitory influence on regional carbon emissions. Notably, the coefficients for the indirect effects surpass those of the direct effects. Moreover, when employing the spatial matrix based on geographical distance, the indirect effect coefficient attains the highest absolute value, reaching 2.977. This suggests that the inhibitory effect of digital trade on carbon emissions has spatial spillover effects, confirming hypothesis 3. Furthermore, this spillover effect is characterized by a “neighborhood effect” greater than the “local effect” and is more dependent on geographical proximity. This phenomenon may be due to several reasons: on one hand, there may be technological spillovers in digital trade activities between regions. Through the exchange of traded products and services between regions, local areas can learn advanced low-carbon technologies and management methods from neighboring regions, thereby optimizing local industries and reducing carbon emissions. On the other hand, the development of digital trade in a region may imply resource sharing and optimization. Some resource-intensive industries may become more specialized in specific regions, while neighboring regions can rely on the output of that region, avoiding the production of the same high-carbon products and thereby reducing carbon emissions in neighboring areas.

## Conclusions and policy recommendations

As China actively explores new avenues for sustainable development, particularly within the framework of the "dual circulation" strategy and the objectives of "carbon peaking" and "carbon neutrality," the significance of digital trade is increasingly evident. To gain a deeper understanding of the pivotal role of digital trade in this context and its implications for carbon emissions, this study utilizes panel data spanning from 2013 to 2021 for 30 Chinese provinces. An index system is devised to assess the levels of regional digital trade development, and panel regression analysis is conducted to investigate the impacts and mechanisms of digital trade on carbon emissions from both supply and demand perspectives. Additionally, spatial econometric models are employed to delve into the spatial spillover effects of digital trade on carbon emissions. The key findings are as follows: (1) The progression of digital trade significantly restrains regional carbon emissions. And this influence is particularly pronounced in the central and western regions of China, as well as in areas characterized by lower levels of carbon emissions. (2) Digital trade holds the potential to stimulate innovation in green technology and improve consumption patterns, thereby facilitating environmentally friendly production practices for businesses and promoting green consumption among residents. (3) The regional expansion of digital trade not only aids in local carbon reduction but also manifests spatial spillover effects, inhibiting carbon emissions in neighboring regions. This "neighborhood effect" surpasses the impact observed at the local level.

In light of these findings, this paper puts forward the following three policy recommendations:

Firstly, actively promote the development of a digital economy with digital trade as the main representative. With the rapid advancement of technology, people's lifestyles have undergone tremendous changes, and the wave of industrial transformation has emerged. Humanity has entered the digital age. We need to focus on the development of digital infrastructure, enhance the digital trade business environment, and boost investment in digital technology research and development. The government can provide financial support and policy guidance, while simultaneously constructing digital trade infrastructure such as cross-border e-commerce platforms and logistics networks. This will help accelerate the integration of digital economies between regions, enhancing the efficiency and feasibility of digital trade.

Secondly, it is necessary to recognize the heterogeneity of digital trade development among different regions. When promoting digital trade development in various provinces, one-size-fits-all approaches should be avoided. Different levels of support should be provided based on the different levels of carbon emissions caused by digital trade, truly achieving tailored policies and narrowing the "digital divide" between provinces. For provinces in the central and western regions with relatively underdeveloped economies and higher levels of carbon emissions, efforts should be made to strongly support the development of local digital trade, promote the tilt of digital resources to these regions, help them leverage their advantages in latecomer development, and maximize the role of digitalization in suppressing carbon emissions. Considering the spillover effects of carbon emissions from digital trade between regions, it is important for regions to establish digital trade cooperation mechanisms to facilitate the sharing of experiences and resources, and to jointly formulate and coordinate efforts to reduce emissions.

Lastly, while promoting the digital transformation of enterprises and green technological innovation, the government also needs to promote green and sustainable consumption among residents. On the one hand, through the establishment of innovation funds to support enterprise digital transformation and providing research and development funds and intellectual property protection, the government can help enterprises enhance their enthusiasm for technological innovation, reduce carbon emissions, and enhance sustainable competitiveness. On the other hand, the government can carry out environmental protection publicity and education activities to increase residents' awareness of sustainable lifestyles. It is important to encourage and support the construction of green consumption infrastructure, such as expanding bike lanes, optimizing public transportation systems, and promoting the supply of green energy, to provide more environmentally friendly choices and reduce the carbon footprint of individuals and families.

## Data Availability

Data sets generated during the current study are available from the corresponding author on reasonable request.

## Appendix 1: Correlation tests of spatial Dubin model

**Table:**

Tests	${\text{W}}_{1}$	${\text{W}}_{2}$	${\text{W}}_{3}$
LM-spatial lag	38.690***	7.076***	17.748***
Robust LM-spatial lag	40.704***	7.544***	16.302***
LM-spatial error	14.602***	6.546**	40.308***
Robust LM-spatial error	16.615***	7.014***	38.861***
LR-spatial lag	37.76***	32.07***	18.75**
LR-spatial error	44.47***	30.74***	15.65**
Wald-spatial lag	39.23***	33.78***	19.68***
Wald-spatial error	47.74***	32.83***	16.26**
Hausman test	-3.74	92.70***	46.69***
LR-ind test	27.39***	33.10***	21.33**
LR-time test	539.98***	584.66***	575.58***

***, **, and * represent statistical significance levels at 1%, 5%, and 10%, respectively.
